# Baicalin alleviates uterine inflammaging and improves eggshell quality in aged laying hens

**DOI:** 10.3389/fimmu.2026.1889930

**Published:** 2026-09-09

**Authors:** Dan Shao, Yuan Qu, Liang Qu, Haibing Tong

**Affiliations:** Jiangsu Institute of Poultry Science, Yangzhou, China

**Keywords:** baicalin, eggshell quality, laying hen, uterine epithelial cell, uterine inflammaging

## Abstract

Aging is a major contributor to the decline in egg quality, with uterine inflammaging playing a key role in this process. Implementing anti-aging strategies to prevent uterine inflammaging and sustain optimal eggshell quality is imperative. This study aimed to characterize age-related changes in the eggshells and uteri of hens at different laying stages and the regulatory effect of baicalin (BAI). Uterine inflammaging became progressively more evident with advancing hen age, as indicated by fibrosis of the lamina propria connective tissue, decreased height and width of primary uterine folds (*P* < 0.05), an increased immunohistochemically positive area for the age-related protein p53, a component of the p53/p21 pathway involved in cellular senescence, elevated expression levels of the proinflammatory cytokines tumor necrosis factor alpha (TNF-*α*) and interleukin-6 (IL-6), and decreased messenger RNA (mRNA) expression of the longevity gene sirtuin 1 (SIRT1) (*P* < 0.05). Additionally, uterine inflammaging was associated with decreased eggshell thickness and breaking strength and with ultrastructural deterioration (*P* < 0.05). Conversely, *in vivo*, BAI treatment alleviated uterine inflammaging, mainly reflected in reduced secretion of the proinflammatory cytokines interleukin-1 beta (IL-1*β)*, IL-6, and interleukin-8 (IL-8) and reduced expression of TNF-*α*, IL-1*β*, p53, and p21 (*P* < 0.05). SIRT1 mRNA expression was significantly increased after BAI treatment (*P* < 0.05). Furthermore, BAI ameliorated eggshell ultrastructure, with fewer type B mammillary knobs and increased effective-layer thickness; it also increased eggshell thickness and breaking strength (*P* < 0.05). *In vitro*, compared to uterine epithelial cells cultured with *β*-galactosidase, BAI pretreatment increased cell viability, decreased the positive staining rate of senescent cells, downregulated the relative expression of IL-6, IL-1*β*, TNF-*α*, p53, and p21, and increased SIRT1 mRNA levels (*P* < 0.05). Overall, uterine inflammaging was closely associated with age-related deterioration in eggshell quality, and BAI administration was associated with improved eggshell quality and reduced uterine inflammaging.

## 1 Introduction

Producing superior-quality eggs is crucial for egg producers to satisfy consumers’ preferences and requirements of the food processing industry (1). As in mammals, laying hens experience a gradual decline in egg production performance with age, particularly a decline in egg quality and an increase in the proportion of soft-cracked eggs (2, 3). Breakage-induced losses during production and transport have been estimated to account for 8-11% of total egg production per year (4), causing substantial economic losses for egg producers and impeding the extension of the laying period (5, 6). Therefore, high-quality eggshells need to be maintained, especially in the late laying period of hens.

The eggshell serves as a protective barrier during embryonic development and against microbial invasion (7), and requires around 20 h to develop in the uterus after ovulation. It is generally acknowledged that uterine health is directly linked to eggshell quality (8), and uterine dysfunction in aging laying hens represents a critical contributor to compromised eggshell quality (9). Evidence indicates that uterine structural deterioration, such as reduced gland density and endometrial fibrosis and atrophy, is associated with decreased mammillary knob density and palisade layer ratio and reduced eggshell breaking strength, thickness, and fracture toughness (10, 11). Furthermore, an experiment conducted in laying hens at three different ages (30, 60, and 72 wk.) revealed that aging leads to poor eggshell formation, accompanied by uterine immune dysfunction, increased crystal size, and widening of the spaces between palisade layers (12). Similarly, other reports have demonstrated that, as laying hens age, cellular senescence and inflammatory responses in the uterus contribute to a decrease in the incidence of early eggshell fusion and an increase in the thickness of the mammillary layer, which together compromise the mechanical properties of the eggshell (9, 13). These studies confirm that eggshell weakening is associated with uterine inflammation and dysfunction caused by aging (14, 15), and this inflammation in the uterus due to aging is known as “inflammaging”. Therefore, in this study, variations in eggshell quality and uterine status with increasing hen age (35, 57, and 100 wk. of age) were analyzed to improve understanding of the relationships among hen age, eggshell quality, and uterine status. Implementing anti-aging strategies to prevent uterine inflammaging and sustain optimal eggshell quality is therefore crucial.

Numerous studies have shown that nutritional interventions may help delay aging and age-related disease (16, 17); in particular, natural polyphenols play an important role in maintaining health during aging (18). Baicalin (BAI), a polyphenolic compound isolated from the roots of *Scutellaria baicalensis Georgi*, has various pharmacological activities, particularly anti-cancer and anti-aging activities attributed to its antioxidant, anti-inflammatory, anti-apoptotic, and anti-excitotoxic effects (19). Interestingly, a study reported that BAI may modulate ovarian granulosa cell viability and improve ovarian function in aged mice (20). Additionally, BAI can alleviate inflammation in aging-periodontitis mice by modulating various inflammatory cytokines, such as interleukin-6 (IL-6), interleukin-1 (IL-1), and tumor necrosis factor-alpha (TNF-*α*) (21). These findings collectively imply that BAI has beneficial effects on inflammaging. However, few studies have investigated the effects of BAI on laying hens, and information on uterine inflammaging is even more limited. Using the abovementioned findings as a framework, we then hypothesized that BAI can ameliorate age-related uterine inflammaging and subsequently improve the eggshell quality. To test this hypothesis, the effects of BAI on uterine inflammaging and eggshell quality in aged hens were analyzed. This study examines, for the first time, the association between uterine inflammaging and eggshell quality decline in naturally aged hens and evaluates baicalin as a potential modulator of reproductive aging.

## 2 Materials and methods

### 2.1 Animals and sample collection for the age-related experiment

A total of 20 healthy young (35 wk. of age), 20 healthy old (57 wk. of age), and 20 older (100 wk. of age) Hy-Line Brown laying hens were randomly selected from a commercial flock (Yangzhong Daqing Poultry Industry Co., Ltd., Zhenjiang, Jiangsu, China). Laying hens were individually housed in single cages (cage size: 23 cm × 38 cm × 39 cm) and exposed to 16 h of light per day at an intensity of 10 lux, with a stable temperature (25 ± 2°C) and humidity (65 ± 5%), following the method described in a previous study (22). All hens were fed the same corn-soybean basal diet (**Table 1**; Jiangsu Yancheng Yuanyao Feed Co., Ltd., Yancheng, Jiangsu, China), with feed and water provided ad libitum in powder form and through nipple drinkers, respectively. After a 1-week adaptation period during which egg-laying time was artificially monitored, 45 eggs (15 eggs per day) were collected over 3 consecutive days to assess the mechanical properties of eggshells. The investigator responsible for eggshell quality measurements was blinded to the age groups. Then, 12 birds from each group were anesthetized by intravenous injection of 50 mg/mL ketamine hydrochloride (50 mg/kg body weight) at 8.5 h post-oviposition, corresponding to the initiation stage of eggshell calcification as described in a previous study (9), and euthanized by cervical dislocation after unconsciousness was achieved. Eggs laid by these 12 birds on the morning of sampling were used to assess the ultrastructure of eggshells. Hens with no egg in the uterus on the day of sampling were excluded, as a developed egg in the uterus was required for accurate staging. No hens died or showed signs of illness during the experiment, and no other exclusion criteria were applied. A block of uterine tissue was fixed in 4% paraformaldehyde for histological and immunohistochemical analyses. The uterine mucosa was collected, frozen in liquid nitrogen, and then stored at −80°C for RNA extraction.

**Table 1:** Composition and nutrient levels of the corn-soybean basal diet (as-fed basis).

Ingredients	Content, %	Nutrient levels^4^	Content, %
Corn	62.5	Metabolizable energy, MJ/kg	11.30
Soybean meal	25.0	Crude protein	16.50
Soybean oil	1.00	Methionine	0.46
Limestone	9.50	Lysine	0.85
CaHPO_4_	1.00	Calcium	3.90
Methionine	0.20	Total phosphorus	0.52
NaCl	0.35	Available phosphorus	0.30
Choline chloride	0.15		
Microelement premix^1^	0.10		
Vitamin premix^2^	0.04		
Compound enzyme^3^	0.16		
Total	100.00		

1 Microelement premix provided the following per kilogram of the diet: copper 8 mg, iron 100 mg, manganese 110 mg, selenium 0.15 mg, iodine 0.35 mg.

2 Vitamin premix provided the following per kilogram of the diet: vitamin A 11,550 IU, vitamin D_3_ 4,375 IU, vitamin E 52.5 IU, vitamin K_3_ 7 mg, vitamin B_1_ 3.5 mg, vitamin B_2_ 10.5 mg, vitamin B_6_ 10.5 mg, vitamin B_12_ 0.035 mg, biotin 0.26 mg, pantothenic acid 21 mg, nicotinic acid 70 mg, folic acid 1.75 mg.

3 Compound enzyme provided the following per gram of the diet: cellulase ≥ 500 IU, xylanase ≥ 25,000 IU, β-glucanase ≥ 2,000 U, mannanase ≥ 2,500 U, amylase ≥ 1,000 U, protease ≥ 8,000 U, pectinase ≥ 1,000 U.

4 Crude protein, calcium, and total phosphorus were measured values, and the rest were calculated values.

### 2.2 Animals and sample collection: BAI-related experiment (*in vivo*)

After the hens were acclimated for 1 week under the same conditions as described in Section 2.1, 40 healthy 85-week-old Hy-Line Brown laying hens (obtained from Yangzhong Daqing Poultry Industry Co., Ltd., Zhenjiang, Jiangsu, China) were randomly assigned to two groups, namely, the CON group and the 20 mg/(kg·d) BAI group. BAI (B95207, purity ≥95%, Beijing Solarbio Science & Technology Co., Ltd, Beijing, China) was dissolved in 5% sodium bicarbonate solution and diluted with sterile physiological saline. Hens in the BAI group were intraperitoneally injected with BAI at 20 mg/(kg·d) at 16:00 for 10 consecutive days, while those in the CON group received an equivalent volume of 5% sodium bicarbonate solution following the same protocol. All injections were performed by one investigator. A total of 49 eggs from the CON group and 50 eggs from the BAI group were collected continuously for 3 days to assess the mechanical properties of eggshells. Thereafter, 15 birds from each group were sacrificed at 8.5 h post-oviposition as described in Section 2.1, corresponding to the initiation stage of eggshell calcification, and eggs laid by these 15 birds on the morning of sampling were used to assess the ultrastructure of eggshells. Hens with no egg in the uterus on the day of sampling were excluded. No mortality or morbidity occurred during the 10-day experimental period, and thus no animals were excluded for health reasons. A block of uterine tissue was fixed in 4% paraformaldehyde for histological and immunohistochemical analyses. The collected uterine mucosa was quickly frozen in liquid nitrogen and subsequently stored at −80°C for enzyme-linked immunosorbent assay (ELISA) and RNA extraction.

### 2.3 Mechanical properties and ultrastructure of eggshells

After the eggs were weighed, the strength of the eggshell was assessed using an egg force reader (EFR-01, ORKA Food Technology, Ltd., Ramat Hasharon, Israel). Next, the contents of the eggs were removed, and the eggshells were washed, dried naturally for 48 h, and weighed to calculate the eggshell ratio. Subsequently, one fragment (~1 cm × 1 cm) was collected from each of the blunt end, equatorial part, and sharp part of the eggshell to measure its thickness using a shell thickness gauge (211-101F, Guilin Guanglu Measuring Instrument Co., Ltd., Guilin, Guangxi, China). Next, two eggshell samples (~0.5 cm × 0.5 cm) from the equatorial part were isolated for cross-sectional ultrastructural analysis and assessment of mammillary body structure, respectively. One piece was directly fixed on the observation table for gold-spraying treatment. Scanning electron microscopy (GeminiSEM 300, Carl Zeiss Microscopy GmbH, Jena, Germany) was performed to observe the cross-sectional ultrastructure at a magnification of ×200, and three points were observed for each eggshell sample. The thickness of the effective and mammillary layers and mammillary knob width were measured using Digimizer 5.4.4.0 software as reported in previous studies (23, 24). The other piece of eggshell was first soaked in a mixed solution (sodium hypochlorite, sodium chloride, and sodium hydroxide) for 24 h to remove the eggshell membranes (25), then treated with gold-spraying and scanned to observe mammillary knob ultrastructural variants according to a previous study (9).

### 2.4 Uterine histomorphology and immunohistochemistry

After fixation with 4% paraformaldehyde for 24 h, the uterus was washed, dehydrated, cleared, embedded in paraffin, and then cut into 5-µm-thick sections. Then, the sections were stained with hematoxylin and eosin (H&E) for histomorphological analysis. The height and width of primary mucosal folds were measured as described in previous studies (15, 26) using a light microscope (CX23, Olympus, Tokyo, Japan) and ImageJ 1.53 software (National Institutes of Health, Bethesda, MD, USA). Additionally, the embedded tissues underwent immunohistochemical (IHC) staining. After deparaffinization and rehydration, the sections were incubated with 3% hydrogen peroxide solution. Subsequently, the sections were washed with phosphate-buffered saline (PBS), blocked with goat serum at room temperature for 20 min, and then incubated with the P53 antibody (80077-1-RR, Proteintech Group, Inc., Rosemont, IL, USA) overnight at 4°C. After the sections were washed with PBS, they were incubated with horseradish peroxidase (HRP)-conjugated secondary antibodies at room temperature for 20 min. The samples were visualized via 3,3′-diaminobenzidine (DAB) staining, and hematoxylin was used for nuclear counterstaining. The percentage of p53 protein-positive area was computed using ImageJ 1.53 software (National Institutes of Health, Bethesda, MD, USA) after images were captured under a microscope (CX23, Olympus, Tokyo, Japan).

### 2.5 Inflammatory cytokine contents of uterine mucosa

Uterine mucosa homogenates were prepared in precooled PBS and centrifuged at 1,000 rpm at 4°C for 10 min. After centrifugation, the liquid supernatant was collected to measure the levels of interleukin-6 (IL-6), interleukin-1 beta (IL-1*β*), interleukin-8 (IL-8), interleukin-10 (IL-10), and interferon-gamma (IFN-γ) with ELISA kits (Shanghai Enzyme-linked Biotechnology Co., Ltd., Shanghai, China), following the manufacturer’s instructions. The following catalog numbers and sensitivities were used: IL-6 (ml059839, 1.56 pg/mL), IL-1β (ml059835, 5 pg/mL), IL-8 (ml059840, 15.62 pg/mL), IL-10 (ml059830, 5.63 pg/mL), and IFN-γ (ml042758, 7.81 pg/mL). The absorbance was measured at 450 nm using a microplate reader (Infinite 200 PRO, Tecan Austria GmbH, Grödig, Austria). Meanwhile, the protein concentrations were quantified with a bicinchoninic acid protein assay kit (A045-4, 20 μg/mL, Nanjing Jiancheng Bioengineering Institute, Nanjing, Jiangsu, China).

### 2.6 RNA isolation and real-time quantitative PCR

Total RNA was extracted from uterine mucosa using TRNzol reagent (Tiangen Biotech (Beijing) Co., Ltd., Beijing, China) following the manufacturer’s instructions, and then reverse-transcribed into single-stranded complementary DNA (cDNA) with the FastKing One-step gDNA Removal Premix (Tiangen Biotech (Beijing) Co., Ltd., Beijing, China). Subsequently, RT-qPCR was conducted using SuperReal Premix Plus (FP205, Tiangen Biotech (Beijing) Co., Ltd., Beijing, China) on a real-time PCR system (StepOnePlus, Applied Biosystems, Foster City, CA, USA). The primers used in this study are listed in **Table 2**. The messenger RNA (mRNA) expression levels of target genes were normalized to *β*-actin and computed using the 2^-ΔΔCt method^.

**Table 2:** Primers used for RT-qPCR.

Genes	Primer sequences (5′ to 3′)	GenBank ID
*TNF-α*	F: GCCCTTCCTGTAACCAGATG	NM_204267.2
	R: ACACGACAGCCAAGTCAACG	
*IL-6*	F: TGCTATGTCAGAGGCGAATGTTG	NM_204628.2
	R: CTGCCATCTGTCACACGGTAAC	
*P53*	F: CTGTACCACGGTGCTGTA	NM_205264.1
	R: AGTGTAAGGATGGTGAGGAT	
*SIRT1*	F: TAGCCAATGGTTTCCACTCC	NM_001004767.2
	R: AAGAATTGTCCGTGGGTCTG	
*IL-1β*	F: ACTGGGCATCAAGGGCTACA	NM_204524.2
	R: GCTGTCCAGGCGGTAGAAGA	
*P21*	F: GGTGGTTATGGAATACTTG	NM_015278599.4
	R: CTGCTATCTGTCCTTCAT	
*β-actin*	F: CAGCCATGTATGTAGCCATCCAG	NM_205518.2
	R: CATCACCAGAGTCCATCACAATACC	

### 2.7 Cell culture and BAI-related experiment (*in vitro*)

The uterine epithelial cells were isolated, cultured, and treated with D-galactose (D-gal) to induce senescence according to the protocol established in our previous study (27). In brief, the uterine epithelial cells from Hy-Line Brown laying hens (30 to 35 weeks old) were isolated via mechanical preparation combined with enzymatic digestion. First of all, the uterine tissue was quickly separated and rinsed with sterile PBS to remove blood after the laying hens were sacrificed. Next, a longitudinal cut was made, and the area was scraped with a glass slide. The fragments of the uterine mucosa were then digested with 0.2% collagenase I (C8140, Beijing Solarbio Science & Technology Co., Ltd., Beijing, China) at 37°C for 35 min. After digestion was terminated with complete culture medium, the digested contents were filtered through 200-mesh cell sieves (Anhui Biosharp Biological Technology Co., Ltd., Hefei, Anhui, China), and the filtrate was centrifuged at 1,500 rpm for 5 min to collect the cells. Then, the cells were resuspended in complete culture medium, and counted using a hemocytometer. After that, these cells were seeded in culture plates coated with Matrigel (356231, Corning Incorporated, Corning, NY, USA) and cultured in complete culture medium containing 10% fetal bovine serum (FBS; Gibco, Carlsbad, CA, USA), 1% penicillin-streptomycin (P1400, Solarbio, Beijing, China), and DMEM/F-12 (C11330500BT, Gibco, Carlsbad, CA, USA) in a 5% CO__2__ incubator at 37°C. After culturing for 24 h, cells were pretreated with BAI at concentrations of 0, 10, 20, 40, 80, 160, 320 μM. After 4 h, the medium was replaced with complete culture medium containing 16 mg/mL D-gal and BAI at the seven concentrations for 24 h, while the cells in the control group were cultured in normal complete medium. After treatment, the cell viability was assessed using Cell Counting Kit-8 (CCK-8) assays (BMU106-CN, Abbkine Biotechnology Co., Ltd., Wuhan, Hubei, China) following the manufacturer’s instructions, and the absorbance was measured at 450 nm using a microplate reader (Infinite 200 PRO, Tecan Austria GmbH, Grödig, Austria). Next, senescence-associated *β*-galactosidase (SA-β-gal) staining (C0602, Beyotime Biotechnology, Shanghai, China) was conducted based on the manufacturer’s instructions, and the image was obtained using a microscope (CX23, Olympus, Tokyo, Japan). The positive rate of *β*-galactosidase staining for cellular senescence was calculated using ImageJ 1.53 software (National Institutes of Health, Bethesda, MD, USA). Moreover, other cells were also harvested for RNA isolation and RT-qPCR analysis.

### 2.8 Statistical analysis

All data were first tested for normality using the Shapiro–Wilk test and for homogeneity of variances using Levene’s test via IBM SPSS Statistics 26.0 software (IBM Corp., Armonk, NY, USA). Then, for experiments involving multiple groups, including the age-related experiment (35, 57, and 100 weeks of age) and the BAI-related *in vitro* experiment (different concentrations of BAI treatment), data were analyzed using one-way ANOVA followed by Tukey’s multiple comparison test. For the BAI-related *in vivo* experiment involving two groups (CON vs. BAI), differences were analyzed using an unpaired Student’s t-test. All data are presented as mean ± SEM. Statistical significance was set at *P* < 0.05, and a tendency was considered when 0.05 < *P* < 0.1. Histograms were generated using GraphPad Prism 8.0.2 (GraphPad Software, San Diego, CA, USA).

## 3 Results

### 3.1 The mechanical properties and ultrastructure of eggshells deteriorated with age

**Figure 1:**
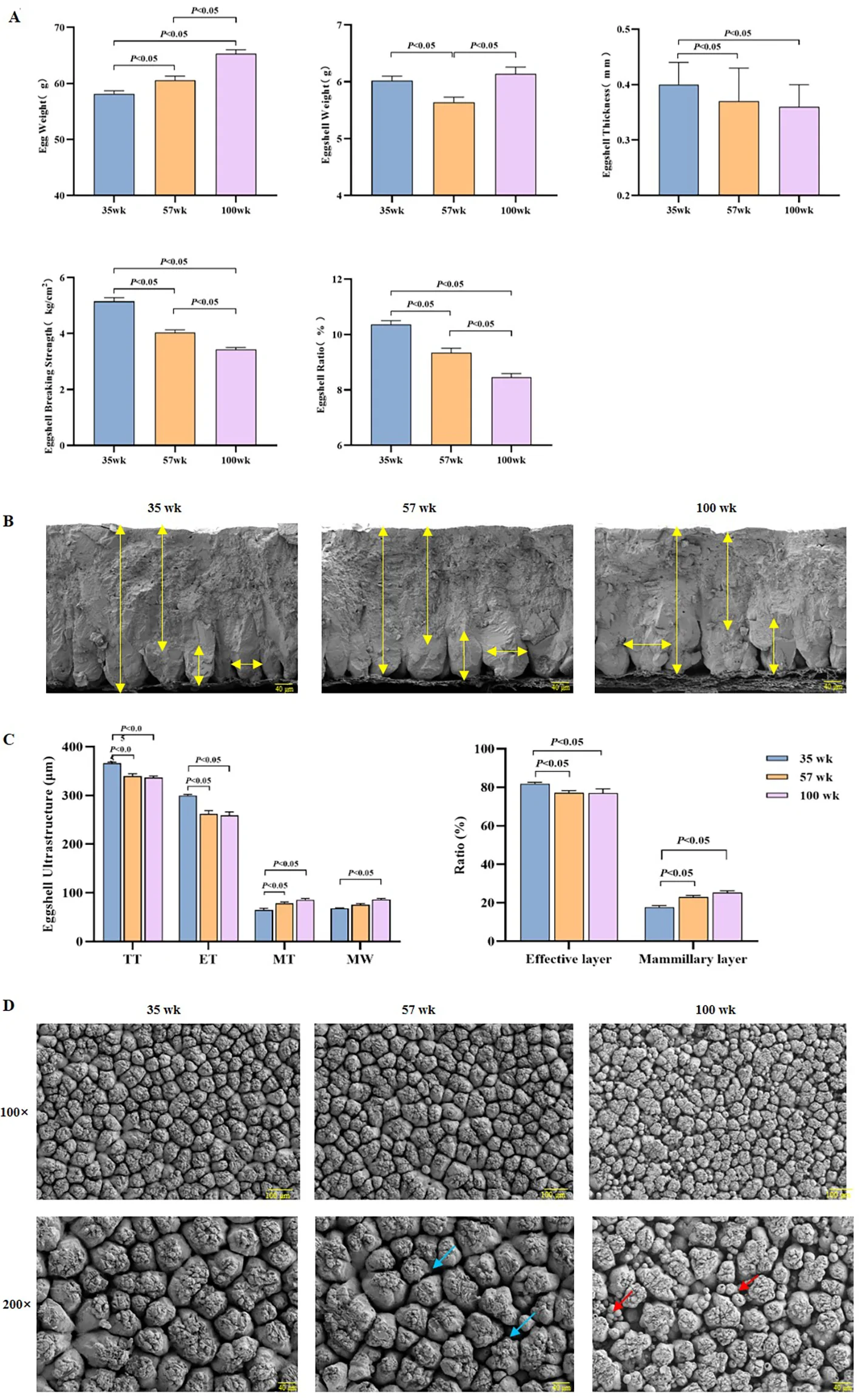
Eggshell mechanical properties and ultrastructure deteriorated with age. **(A)** Eggshell mechanical properties (n = 45). **(B)** Image of eggshell cross-sectional ultrastructure under a scanning electron microscope (magnification, ×200). **(C)** Results of the eggshell ultrastructure characteristics (n = 12). TT, total thickness; MW, mammillary width; ET, effective layer thickness; MT, mammillary layer thickness. **(D)** Scanning electron microscopy image of eggshell inner-surface ultrastructure. Blue arrow, late fusion; red arrow, type B mammillary knob. All data were represented as mean ± SEM. *P* < 0.05 indicates a significant difference between groups, and 0.05 < *P* < 0.1 indicates a tendency toward a difference between groups.

The results for eggshell mechanical properties are shown in **Figure 1A**. Eggshell ratio, eggshell thickness, and eggshell breaking strength decreased significantly as the laying hens became older (*P* < 0.05), whereas egg weight and eggshell weight significantly increased (*P* < 0.05). Regarding eggshell ultrastructure (**Figures 1B–D**), the thickness and ratio of the eggshell effective layer were significantly lower in the 57- and 100-week-old hens than in the 35-week-old hens (*P* < 0.05). As hens grew older, the thickness and ratio of the mammillary layer, along with the width of the mammillary knobs, increased in eggshells (*P* < 0.05). Moreover, the gaps between the adjacent mammillary knobs became wider as the age of the hens increased. The type B bodies occurred in the eggshell mammillary layer of 57- and 100-week-old hens, with a higher abundance observed in the 100-week-old hens; however, they were not found in the 35-week-old hens.

### 3.2 Aging induced uterine damage and inflammation

**Figure 2:**
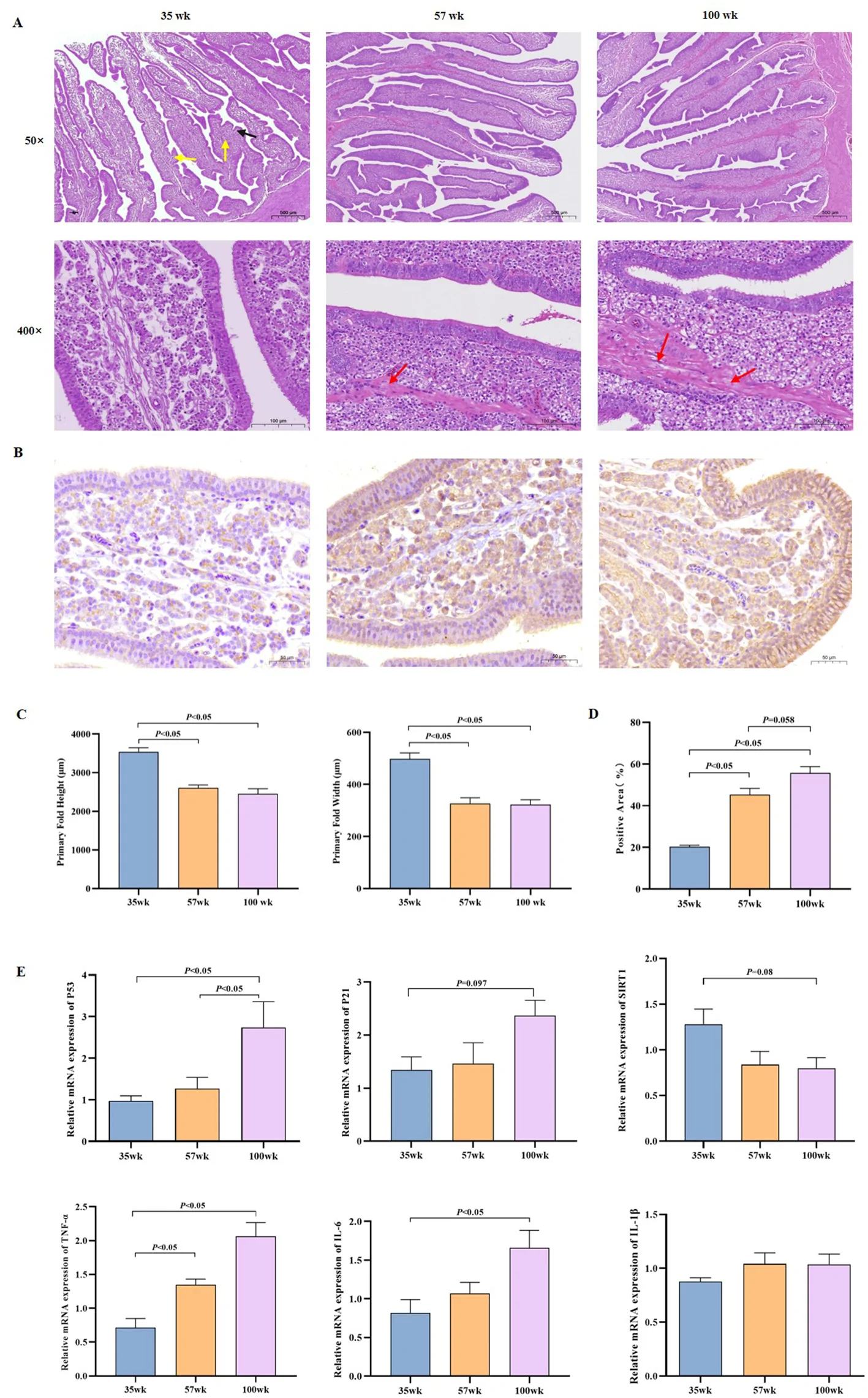
Aging was associated with uterine damage and altered mRNA expression of genes related to inflammaging in the uterus of laying hens. **(A)** Representative Hematoxylin and eosin (H&E) staining images of the uterus of laying hens at 35, 57, and 100 weeks (magnification, ×50 and ×400). **(B)** Representative immunohistochemical staining images with p53 antibody visualized using chromogen diaminobenzidine (brown staining) in the uterus of laying hens at 35, 57, and 100 weeks (magnification, ×400). **(C)** Morphology parameters included height and width of primary uterine folds in the uterus of laying hens at 35, 57, and 100 weeks (n = 12). **(D)** Percentage of p53 protein-positive area in the uterus of laying hens at 35, 57, and 100 weeks (n = 12). **(E)** The mRNA expression of genes related to immune response and aging in uterine mucosa at 35, 57, and 100 weeks (n = 12). Yellow arrow, secondary folds; black arrow, tertiary folds; red arrow, lamina propria connective tissue. All data were represented as mean ± SEM. *P* < 0.05 indicates a significant difference between groups, 0.05 < *P* < 0.1 indicates a tendency toward a difference between groups.

H&E staining showed significant variations in uterine structure among the three age groups (**Figures 2A, C**). The height and width of primary uterine folds in the uterus of 57- and 100-week-old hens were significantly lower than those in the 35-week-old hens (*P* < 0.05). The uteri of the 35-week-old hens showed adequate development, with longer mucosal folds branching into primary, secondary, and tertiary folds. As the hens became older, the number and length of cilia in the uterus decreased slightly in the 57- and 100-week-old hens, while the degree of fibrosis of the lamina propria connective tissue increased, with pronounced mucosal fold atrophy observed in 100-week-old hens. Consistently, the results of the immunohistochemical analysis indicated that aging was associated with a significantly higher percentage of p53 protein-positive area in the uterine tissue (*P* < 0.05; **Figures 2B, D**), and the epithelial cells and tubular glands of the uterus exhibited abundant cytoplasmic particle staining in the 57- and 100-week-old hens. Besides, the results of RT-qPCR analysis showed that aging significantly increased the mRNA expression of TNF-*α*, IL-6, and p53, but significantly decreased SIRT1 mRNA expression (*P* < 0.05, **Figure 2E**). Additionally, aging tended to increase the mRNA expression of p21 (P = 0.097, **Figure 2E**).

### 3.3 BAI improved eggshell mechanical properties and ultrastructure

**Figure 3:**
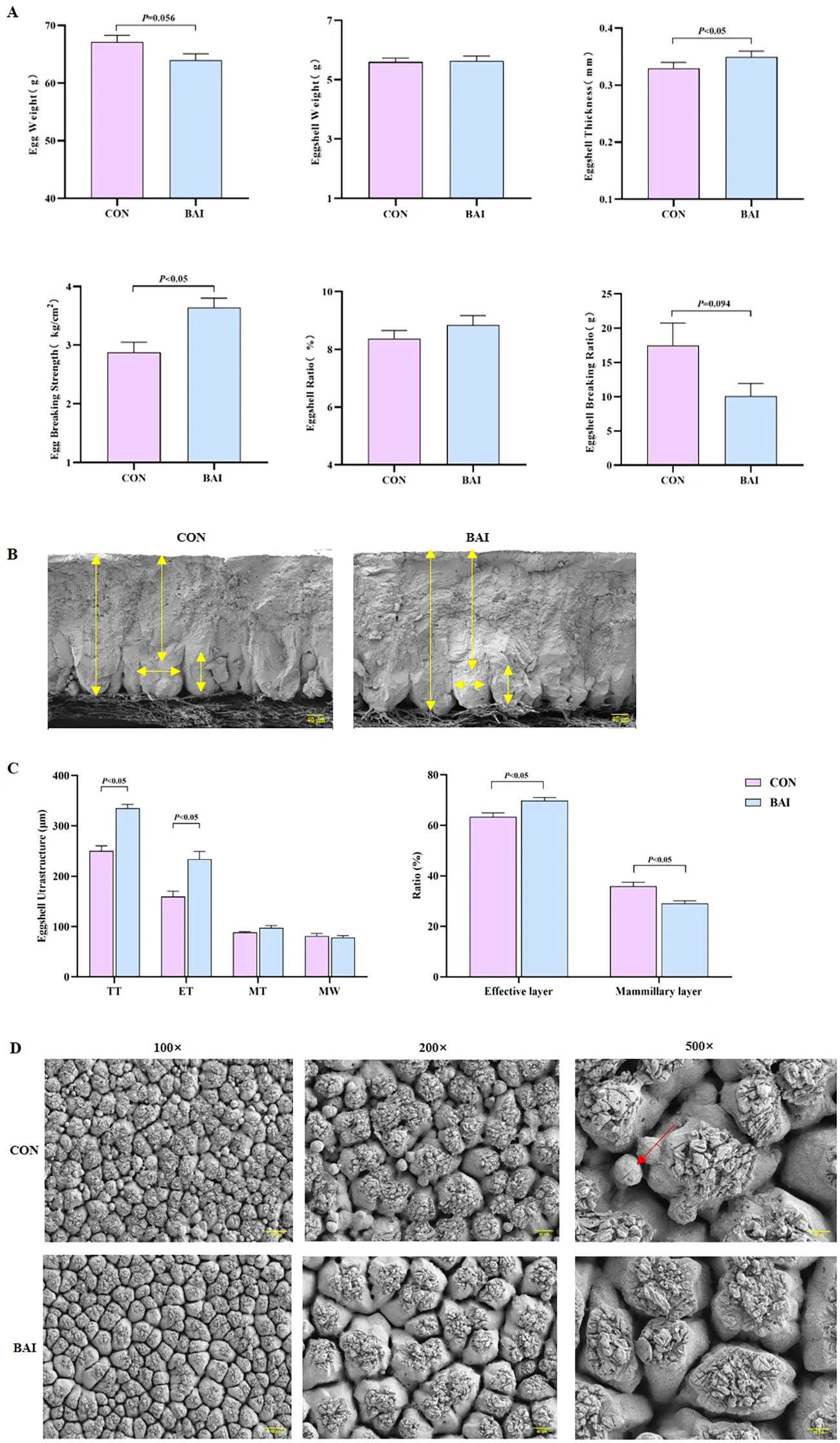
BAI modulated the mechanical properties and ultrastructure of eggshells. **(A)** Eggshell mechanical properties in the control and BAI groups (n = 49 and 50, respectively). **(B)** Image of eggshell cross-sectional ultrastructure in the control group and the BAI group. **(C)** Results of the eggshell ultrastructure characteristics (n = 15). TT, total thickness; MW, mammillary width; ET, effective layer thickness; MT, mammillary layer thickness. **(D)** Scanning electron microscopy image of eggshell inner-surface ultrastructure. Red arrow, type B mammillary knob. All data were represented as mean ± SEM. *P* < 0.05 indicates a significant difference between groups, 0.05 < *P* < 0.1 indicates a tendency toward a difference between groups.

As illustrated in **Figure 3A**, there was an increase in eggshell breaking strength and thickness in response to BAI (*P* < 0.05). Besides, BAI tended to decrease the breakage rate (*P =* 0.094). However, egg weight, eggshell weight, and eggshell ratio were not affected by BAI treatment (*P* > 0.05). With regard to eggshell ultrastructure (**Figures 3B–E**), BAI significantly elevated the total thickness of the eggshell, the thickness and ratio of the effective layer, and the ratio of the mammillary layer (*P* < 0.05). The distribution of mammillary knobs was more symmetrical, and type B mammillary knobs were fewer in the BAI group than in the control group.

### 3.4 BAI ameliorated uterine damage and inflammation in aged hens

**Figure 4:**
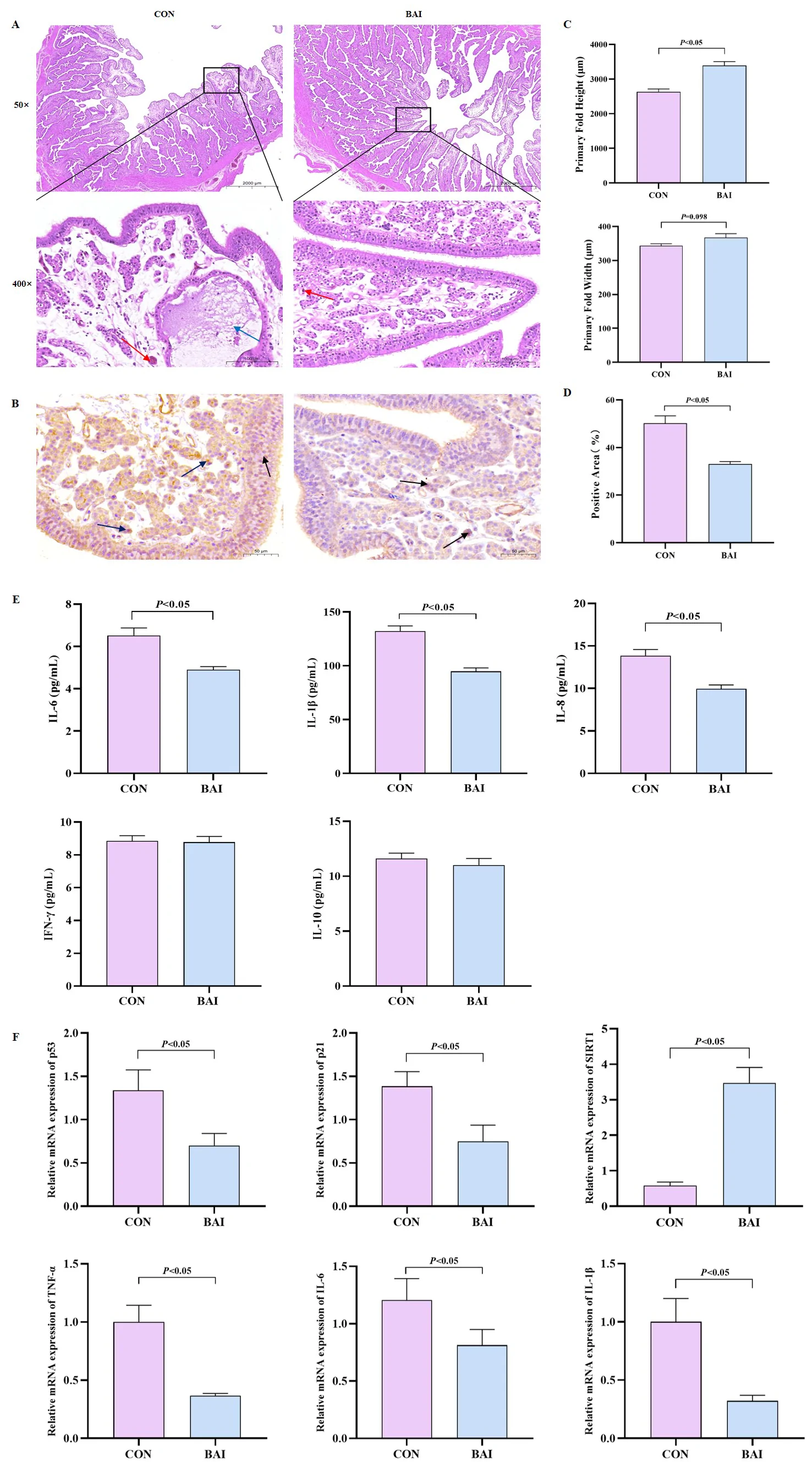
BAI regulated uterine morphology and mRNA expression of genes related to inflammaging in the uterus of laying hens. **(A)** Hematoxylin and eosin (H&E) staining images of the uterus from laying hens in the control and BAI groupss. **(B)** Representative immunohistochemical staining images with p53 antibody in the uterus of laying hens in the control and BAI group. **(C)** Morphology parameters included height and width of primary uterine folds in the uterus from laying hens in the control and BAI groups (n = 15). **(D)** Percentage of p53 protein-positive area in the uterus of laying hens in the control and BAI groups (n = 15). **(E)** The contents of proinflammatory cytokines in uterine mucosa from laying hens in the control and BAI groups (n = 15). **(F)** The mRNA expression of genes related to immune response in uterine mucosa from laying hens in the control and BAI groups (n = 15). Blue arrow, serous substances in the lumen of the tubular glands; black arrow, higher p53-positive staining; red arrow, mild congestion of the vessels in the lamina propria and muscular layer. All data were represented as mean ± SEM. *P* < 0.05 indicates a significant difference between groups, 0.05 < *P* < 0.1 indicates a tendency toward a difference between groups.

Following BAI injection (**Figure 4**), the height of primary uterine folds increased significantly (*P* < 0.05), and their width tended to increase (*P* = 0.098). Further observation showed the structures of the uterine mucosa, muscle layer, and outer membrane were clearly distinguishable in both control and BAI groups, with no obvious fibrosis, although mild vascular congestion was observed in the lamina propria and muscular layers of the mucosa in both control and BAI groups. Meanwhile, BAI appeared to ameliorate the cystic changes in the lamina propria and lessen the serous material in the glandular cavities. Besides, the results of the uterine immunohistochemistry analysis showed that BAI could significantly reduce the percentage of p53 protein-positive area in the uterus (*P* < 0.05). Additionally, BAI treatment significantly inhibited the secretion of proinflammatory cytokines IL-1*β*, IL-6, and IL-8 in uterine mucosa (*P* < 0.05). Meanwhile, a significant decrease in the relative mRNA expression of TNF-*α*, IL-1*β*, p53, and p21, and an increase in the relative mRNA expression of SIRT1 were recorded in the BAI group (*P* < 0.05).

### 3.5 BAI exhibits anti-inflammaging effect on uterine epithelial cells treated with D-gal

**Figure 5:**
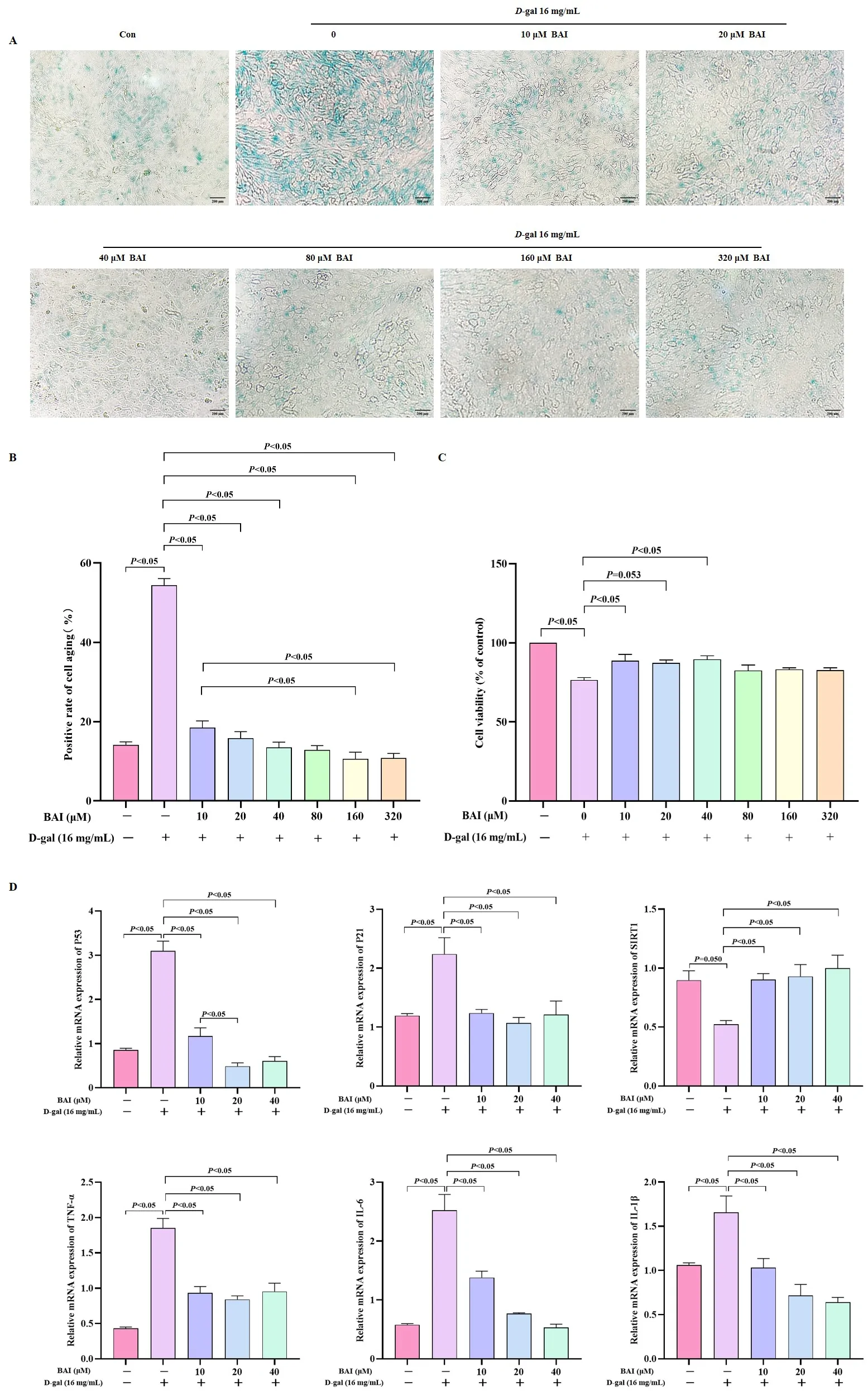
BAI exhibited an anti-inflammaging effect on uterine epithelial cells treated with D-gal. **(A)** Images of cellular senescence detected by SA-*β*-gal staining after treatment with D-gal and different concentrations of BAI. **(B)** The positive rate of SA-*β*-gal in uterine epithelial cells treated with D-gal and different concentrations of BAI (n = 6). **(C)** The viability of uterine epithelial cells treated with D-gal and different concentrations of BAI (n = 6). **(D)** The mRNA expression levels of proinflammatory cytokines (*IL-6*, *IL-1β*, and *TNF-α*) and *p53*, *p21*, and *SIRT1* in uterine epithelial cells treated with D-gal and different concentrations of BAI (n = 6). All data were represented as mean ± SEM. *P* < 0.05 indicates a significant difference between groups, and 0.05 < *P* < 0.1 indicates a tendency toward a difference between groups.

To determine whether BAI could ameliorate senescence-related changes in uterine epithelial cells, SA-*β*-gal staining was used to identify senescent cells, with blue-stained areas indicating senescence. As shown in **Figures 5A, B**, D-gal significantly increased the positive staining rate of senescent cells (*P* < 0.05), and the positive rate of senescent cells decreased in a dose-dependent manner following BAI treatment (*P* < 0.05). Conversely, cell viability decreased with D-gal treatment, whereas BAI increased cell viability (*P* < 0.05, **Figure 5C**). Furthermore, compared with the D-gal group, BAI addition downregulated the relative mRNA expression of IL-6, IL-1*β*, TNF-*α*, p53, and p21 in uterine epithelial cells (*P* < 0.05). Meanwhile, BAI treatment resulted in higher SIRT1 mRNA expression than in the D-gal group (*P* < 0.05).

## 4 Discussion

The age-related decline in eggshell quality is a critical issue for laying hens during the late stages of poultry production (28). A recent study evaluated eggshell quality from hens that were 300, 500, and 700 days old and showed that both eggshell thickness and strength declined as the hens got older (29). Analogously, our results showed a significant progressive reduction in these traits, which might increase the risk of breakage during egg collection and transport (30). Besides, the age-associated impairment of eggshell mechanical properties was considered to be associated with deterioration in eggshell ultrastructure (9). In the current study, eggshells from aged hens displayed marked ultrastructural deterioration, notably in the mammillary layer, which was characterized by obvious structural defects, for example, the damaged mammillary caps and a higher frequency of structural variations including type B bodies. This variability in ultrastructure was also observed in Xinyang blue-shelled laying hens (29), suggesting that it is a common pattern in aged hens. As mentioned in a previous report, the effective layer was the main ultrastructural feature determining the mechanical characteristics of eggshells, and a decrease in its thickness could compromise eggshell thickness and strength, leading to a higher incidence of breakage (31). In addition, the mammillary layer, where cracks initially develop (32), is also the main structure that determines the mechanical properties of eggshells (33). The external force acting on the eggshell is generally concentrated at the site of the knob and propagates along the knob gap (34). When the thickness of the mammillary layer increased, the depth of external force propagation increased; together with wider gaps between adjacent mammillary knobs, which decreased the binding force between the mammillary knobs and the eggshell membrane, these changes increased the likelihood of impairment of eggshell mechanical properties (12). Abnormal structures such as type B bodies and disordered mammillary knob alignment in eggshells became more prevalent as the hens got older (9). Although the type B bodies were deemed not to contribute to the fracture resistance, they weakened the base of the palisade layer (35). These findings further revealed that hen age was associated with structural variations and deterioration in eggshell quality, particularly at 100 weeks of age, an extreme stage critical for understanding uterine inflammaging and supporting the industry’s move toward extended 100-week laying cycles.

A key factor governing eggshell quality is the healthy structure and function of the oviduct, especially the uterine section, where the eggshell develops (36). As hens get older, uterine morphology undergoes damage, manifested as interstitial edema, loose connective tissue with a few inflammatory cell infiltrations (23), histological deformation, fibrosis, atrophy, and loss of ciliated cells (12, 37); these changes impair the uterine capacity to secrete the minerals and proteins required for shell formation (38). Similarly, in the present study, aging reduced the height and width of primary uterine folds, decreased cilia number and length, and increased fibrosis of the lamina propria connective tissue and mucosal fold atrophy, implying a hindered regenerative capacity of uterine cells (39). To better assess age-associated changes in the uterus, we evaluated the classical p53/p21 senescence pathway. p53, serving as an indicator for assessing the health status of the uterus (40), and its downstream effector p21 (41) were upregulated with age, suggesting uterine senescence that may compromise eggshell formation (37). Conversely, SIRT1 expression declined in aged hens. As a member of the sirtuin family, SIRT1 activation has been demonstrated to reduce apoptosis and ameliorate mitochondrial damage and ovarian aging (42, 43), and its age-related decline may reflect loss of transcriptional activation (e.g., PGC-1α, HIF-1α) involved in mitochondrial biogenesis and lifespan extension, ultimately increasing the expression of target genes involved in senescence and inflammation (44). Concurrently, aging increased uterine TNF-α and IL-6 expression. Accumulating evidence indicates that aging is accompanied by inflammation (9, 45), and under such circumstances, inflammatory cytokine activity might increase, and an inflammatory microenvironment might develop in senescent uterine cells (46). IL-6, a key senescence-associated secretory phenotype (SASP) factor (47), was shown to decrease the content of calcium-binding protein D28K (CaBP-D28K) in the uterus, thereby disrupting the Ca^2+^ transport for eggshell formation (48). Additionally, TNF-α is recognized as a key regulator of pro-inflammatory cytokine production throughout the aging process, and elevated TNF-α expression could suppress the mRNA expression of osteopontin and calbindin-1 (CALB1) in uterine mucosa, leading to structural deterioration of the eggshell (49). Overall, these observations suggested that aging was associated with uterine inflammation, dysfunction, and senescence, which might collectively impair eggshell formation capacity. Thus, it makes sense to conclude that mitigation of age-associated inflammation might be a strategy to maintain uterine integrity and enhance eggshell quality in hens during the late laying period.

Baicalin (BAI), one of the most abundant natural flavonoid glycosides, has beneficial biological activities, especially the modulation of anti-inflammatory effects (50, 51). Although BAI has been shown to ameliorate avian pathogenic *Escherichia coli* (APEC), *Clostridium perfringens*-induced intestinal injury in chicks (52, 53), and alleviate thiram-induced tibial dyschondroplasia of broilers (54), its effects on aged laying hens remained unexplored. In this study, BAI administration was associated with improved eggshell ultrastructure and mechanical properties, consistent with a previous report (55). Meanwhile, BAI treatment was associated with reduced inflammatory cytokines, suppression of p53/p21 senescence markers, and elevated SIRT1 expression in uterine mucosa, paralleling observations in BAI-fed aged rats (51). Interestingly, this study showed reduced levels of the proinflammatory cytokines IL-1β, IL-6, and IL-8, but no reduction in IL-10 or IFN-γ, suggesting a preferential action of BAI on the NF-κB-driven proinflammatory cascade (56). These findings suggest a correlative relationship between BAI, attenuated uterine inflammaging, and improved eggshell quality. However, causality cannot be inferred, and the 10-day treatment period may be insufficient to fully reverse chronic uterine aging. Longer-term studies are therefore needed to validate the sustained effects of BAI on uterine health and eggshell quality.

To better assess the anti-inflammaging role of BAI, we examined its role in D-galactose-induced senescent uterine epithelial cells. As the most abundant cell type in the uterus (57), uterine epithelial cells not only produce and secrete shell matrix proteins but also actively mediate the transport of various ions (58), contributing to eggshell formation. Although studies on the function of BAI in uterine epithelial cells are extremely limited, similar studies have also shown that BAI suppresses inflammation in epithelial cells. For example, BAI was reported to mitigate lipopolysaccharide (LPS)-induced inflammation of porcine intestinal epithelial cells (59), and attenuate H_2_O_2_-driven inflammation and oxidation of bovine mammary epithelial cells by regulating inflammatory cytokine levels (60). Analogously, our *in vitro* results corroborated the *in vivo* findings, demonstrating that BAI attenuated D-gal-induced cellular senescence, as evidenced by reduced SA-β-gal activity, suppression of the p53/p21 pathway, and decreased inflammatory cytokine expression, while increasing SIRT1 mRNA levels. This consistency across *in vivo* and *in vitro* models strengthens the association between BAI and reduced uterine inflammaging. Given that eggshell matrix proteins have been shown to regulate calcite crystal nucleation and growth (61, 62), which are critical for proper eggshell formation, future investigations should examine whether the beneficial effects of BAI on eggshell quality are mediated through the regulation of these specific matrix proteins. Such investigations would provide a more complete biological explanation for the observed improvements in eggshell ultrastructure and mechanical properties.

## 5 Conclusion

**Figure 6:**
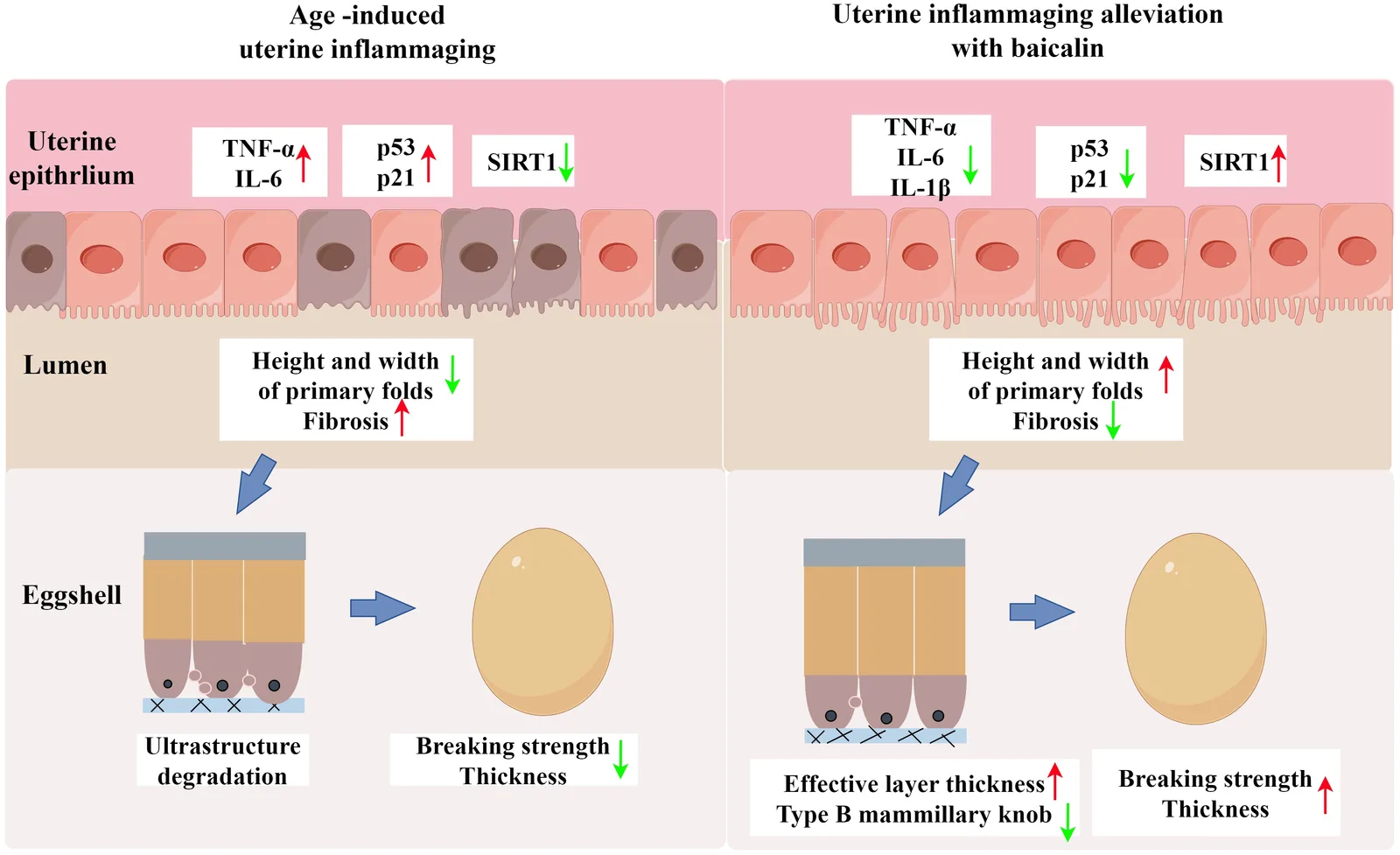
A schematic diagram of the potential regulatory network of uterine inflammaging-mediated eggshell quality deterioration and the regulatory effect of baicalin (BAI).

In summary, the uterine inflammatory status was associated with weakened ultrastructure and mechanical properties of eggshell with aging, implying a crucial role of inflammatory status in uterine functions involved in eggshell formation. BAI treatment was associated with improved uterine morphology, modulation of uterine inflammatory status, reduced uterine senescence, and enhanced eggshell ultrastructure and mechanical properties in aged laying hens (**Figure 6**). This study showed that baicalin treatment was associated with reduced uterine inflammaging and improved eggshell quality in aged laying hens, providing a correlative framework for understanding age-related declines in eggshell quality.

## Data Availability

The raw data supporting the conclusions of this article will be made available by the authors, without undue reservation.
